# Misdiagnosed Progressive Multifocal Leukoencephalopathy (PML) in an HIV-Negative Patient With Discoid Lupus: A Case Report

**DOI:** 10.7759/cureus.42030

**Published:** 2023-07-17

**Authors:** Aliaa Mousa, Muhammad Humayoun Rashid, Kudret Arslan, CamelLia Nabati Lofrese, Nazish Najeeb

**Affiliations:** 1 Internal Medicine, Capital Health Regional Medical Center, Trenton, USA; 2 Internal Medicine, Nishtar Medical University, Multan, PAK; 3 Emergency Medicine, Capital Health Medical Center, Trenton, USA

**Keywords:** progressive multifocal leukoencephalopathy, adoptive transfer of anti-polyomavirus specific t cells, immune checkpoint inhibitors (icis), discoid lupus erythematosus (dle), cerebro-vascular accident (stroke), hiv-negative progressive multifocal leukoencephalopathy

## Abstract

Progressive multifocal leukoencephalopathy (PML) is a rare fetal disease that has been uprising since the 1980s. Accurate diagnosis can be challenging and requires a thorough clinical suspicion, particularly among individuals who do not have HIV infection. Further diagnostics studies including cerebrospinal fluid analysis are required for DNA polymerase chain reaction (PCR) and if negative, more invasive tests like Brain biopsy are required. Herein, we describe a rare case of a 64-year-old female with a history of discoid lupus for 30 years who was not on any medications and presented to the hospital multiple times with different neurological deficits. The initial diagnosis consistently pointed toward a stroke until a critical turning point when a cerebrospinal fluid sample tested positive for John Cunningham (JC) virus DNA. Unfortunately, by the time the disease was identified, it had already progressed significantly, resulting in the unfortunate demise of the patient.

To our knowledge, this represents the second reported case of PML in a patient with discoid lupus who lacks other commonly observed risk factors for the disease. This finding underscores the significance of maintaining clinical attentiveness within this specific patient population.

## Introduction

Progressive multifocal leukoencephalopathy (PML) is a subacute demyelinating infection of the central nervous system (CNS) caused by the polyomavirus John Cunningham (JC) [1]. This opportunistic infection primarily affects oligodendrocytes, with the virus remaining latent in the kidneys and lymphoid tissue after asymptomatic primary infection in childhood [2]. However, in individuals with profound cellular immunosuppression, the virus can reactivate and spread to the CNS.

PML is predominantly observed in immunosuppressed patients, including those with AIDS (79%), hematologic malignancies (13%), organ transplant recipients (5%), and individuals with autoimmune diseases undergoing immunosuppressive therapy (3%) [3]. Occasional cases have been reported in patients with occult immunosuppression, such as hepatic cirrhosis and renal failure. The introduction of highly active antiretroviral therapy (HAART) has led to a decrease in the incidence and mortality of PML [4].

## Case presentation

**Figure 1:**
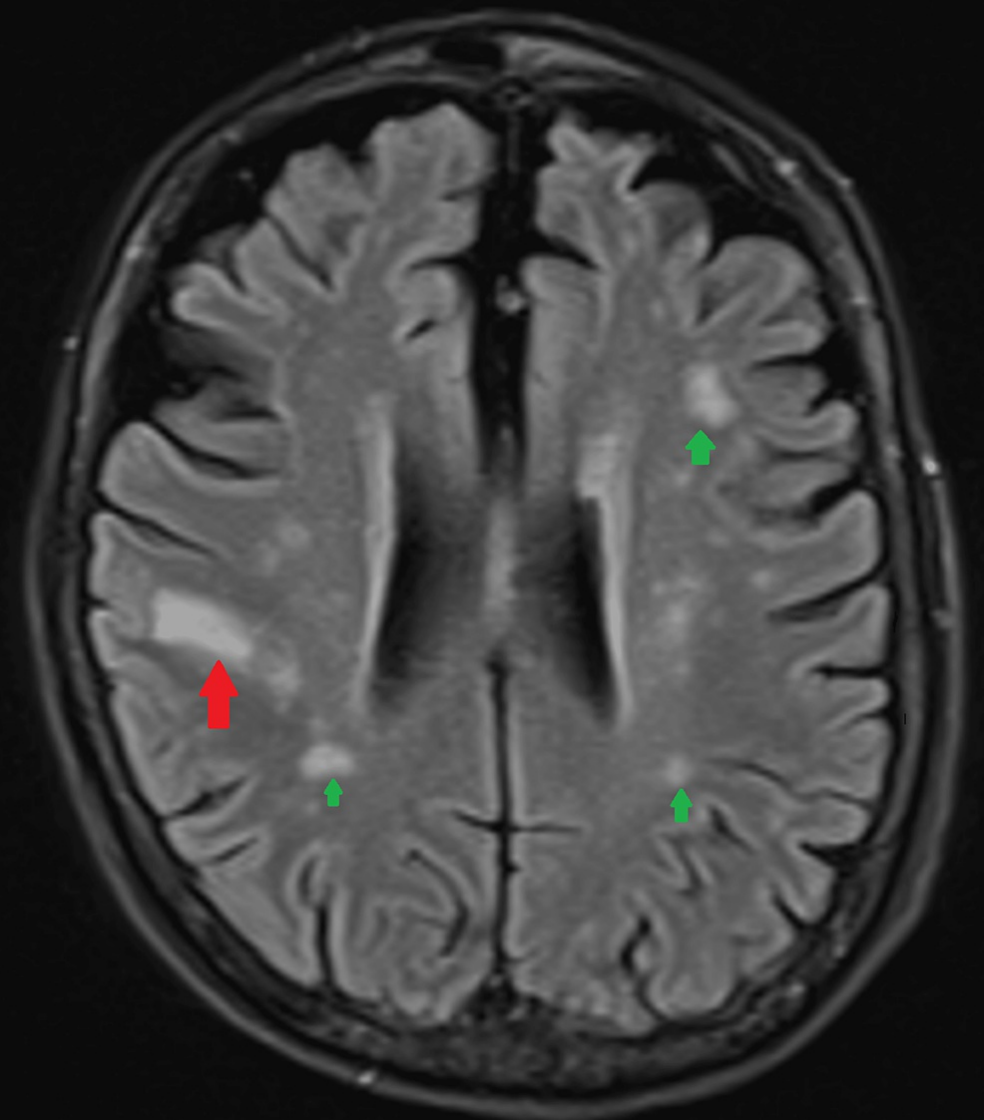
Red arrow showing restricted diffusion within the right parietal lobe, along the postcentral gyrus, with local mass effect, in keeping with acute/subacute infarction. Green arrows showing multiple additional foci of hyperintensity within the bilateral corona radiator/centrum semiovale suggestive of subacute infarctions in watershed pattern.

The patient that we encountered is a 64-year-old female with a past medical history of heart failure with reduced ejection fraction (HFrEF), discoid lupus not on any immunosuppressant, hypertension, hyperlipidemia, and duodenal angiodysplasia presented to the emergency department in September 2022 with severe dizziness and unsteady gait. Magnetic resonance imaging (MRI) of the brain (Figure 1) revealed restricted diffusion within the right parietal lobe with local mass effect suspected of acute/subacute infarction. She was started on antiplatelet therapy with clopidogrel 75 mg and atorvastatin 40 mg for secondary prophylaxis. Dual antiplatelet therapy was not given due to the high risk of bleeding in the setting of duodenal angiodysplasia.

**Figure 2:**
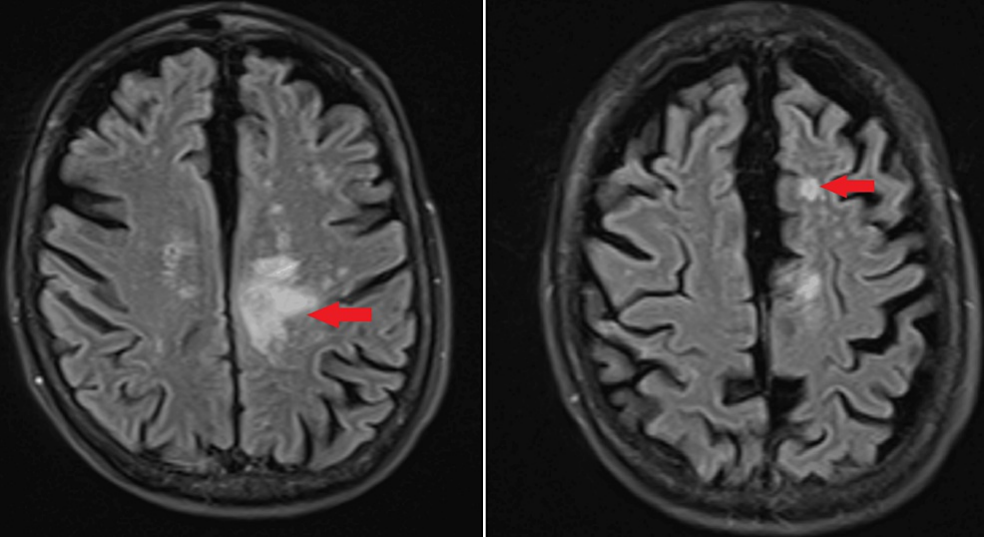
Red arrows show new prominent signals in the posterior left frontal lobe involving cingulate gyrus and body of corpus callosum without associated mass effect or abnormal enhancement compatible with subacute infarction versus progressive multifocal leukoencephalopathy (left and right).

Six months later in March 2023, the patient presented again with worsening unsteadiness and frequent falls. She stated that the unsteadiness has been gradually worsening for the past two months and is associated with generalized weakness more pronounced on the right side than the left side. Motor strength in the right lower extremity was 2/5. No other motor deficits. No sensory or no cranial nerve deficit was noticed. No cerebellar signs were present. Repeat MRI brain (Figure 2) showed an abnormal signal in the posterior left frontal lobe involving the cingulate gyrus and body of the corpus callosum without associated mass effect or abnormal enhancement compatible with subacute infarction versus PML. An MRI of the head and neck was done that showed no occlusion of the arteries within the brain and the neck. Trans-esophageal echocardiogram ruled out any thrombus in the heart or patent foreman ovale. Hypercoagulable workup was negative. Lumbar puncture was advised to look for infectious and inflammatory etiologies, but the patient refused. The patient was discharged to inpatient rehab with a 30-day cardiac monitor to rule out any cardiac event as a cause of recurrent stroke. She was also instructed to have a malignancy work-up done outpatient given her history of smoking, weight loss, and recurrent stroke.

**Figure 3:**
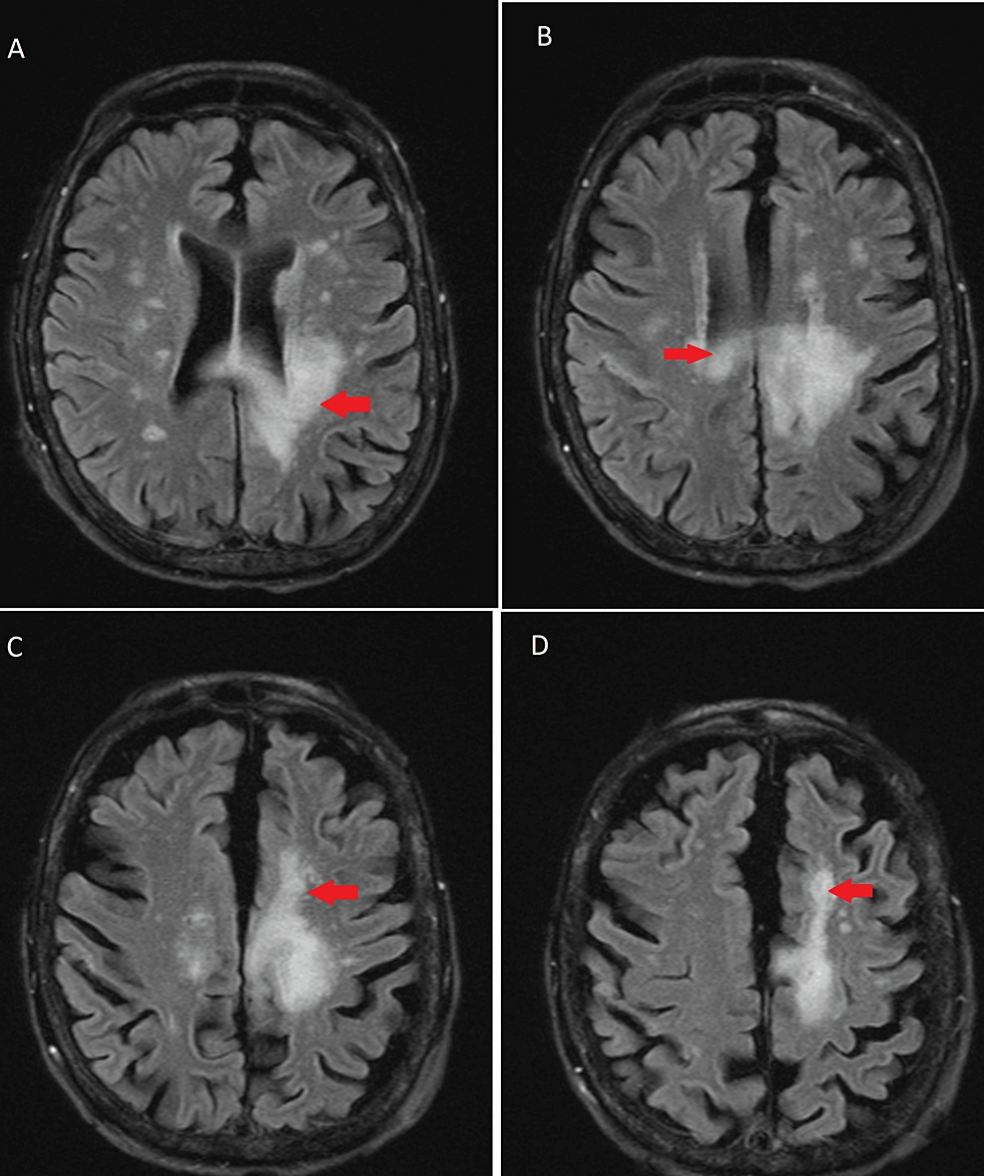
Red arrows show infiltrative hyperintense signal within the left parietal (A), left frontal (C, D), and left temporal lobe involving the cingulate gyrus extending across the body of the corpus callosum to the right frontal lobe (B).

In April 2023, she presented again with altered mental status, worsening weakness, staring spells, and repetitive hand movements. The neurologist recommended a repeat MRI brain with contrast (Figures 3A-3D) and MR spectroscopy and 24-hour video EEG. The patient was started on levetiracetam 500 mg twice daily for seizure prophylaxis. Electroencephalography showed left frontotemporal focal slowing and sharp waves suggestive of underlying stroke or inflammation leading to that epileptiform activity. MRI brain showed a hyperintense signal within the left frontal, parietal, and temporal lobes, involving the cingulate gyrus, extending across the body of the corpus callosum to the right frontal lobe. No significant associated ADC reduction or susceptibility. At this time differential diagnosis included tumefactive multiple sclerosis (MS), PML, and infectious or autoimmune etiology. The family refused to get the lumber puncture done. MS workup including MRI cervical and thoracic spine was negative, showing no enhancing abnormality or abnormal signal within the spinal cord. Malignancy workup including CT chest abdomen and pelvis and HIV was negative. Autoimmune work including anti-neutrophil cytoplasmic antibodies (ANCA), IgG, IgA, IgM, and serum protein electrophoresis (SPEP) with immunofixation (IFE) was normal as well. Considering tumefactive MS as the top priority diagnosis, the patient was started on 1g IV Methyl prednisone daily and plasmapheresis to look for a response. The patient was deteriorating clinically and the family finally agreed to a lumbar puncture. Cerebrospinal fluid (CSF) cytology showed no malignant cells but numerous lymphocytes. Meningitis/encephalitis panel negative. CSF and blood cultures were negative as well. Polymerase chain reaction (PCR) for the JC virus (JCV) came back positive. The diagnosis was made as PML caused by the JCV. Steroids and plasmapheresis were stopped. The family was offered experimental treatment vs hospice care. They agreed to proceed with hospice and the patient died later on in the hospital.

## Discussion

Based on demographic characteristics and comorbid associations, PML can be classified into three distinct periods**.** Initially described in 1958, the viral etiology of PML was discovered in 1971, leading to the naming of the virus as the JCV [1]. In the first two decades following its discovery, PML was primarily associated with hematologic malignancies, systemic inflammatory diseases treated with immunosuppressive medications, and organ transplant recipients. The HIV pandemic in the 1980s significantly increased the incidence of PML, making it an AIDS-defining condition. However, the advent of combination antiretroviral therapy (cART) has resulted in a decline in HIV-associated PML [2].

The third phase of PML emerged with the introduction of new immunomodulatory therapies, particularly natalizumab, an alpha-4 integrin antagonist used for MS and Crohn's disease treatment. Natalizumab poses the highest risk for PML. Iatrogenic cases of PML were first discovered in 1997 with rituximab in the context of non-Hodgkin lymphoma. Currently, the FDA drug list includes 20 medications known to increase the risk of PML [3].

The risk of PML among immunocompromised patients shows variation in different populations, indicating the involvement of additional factors that modify the risk [4]. The pathogenesis of PML can be influenced by viral mutations, which can increase neurovirulence. Certain variants, especially in the VP1 protein, have been linked to higher susceptibility or more aggressive forms of the disease [5]. Furthermore, host genetic risk factors have been identified. For instance, individuals positive for the HLA-DRB1*0401 allele may have a limited ability to mount antiviral responses. Polymorphisms of the tumor suppressor protein p53, which acts as a binding site for the large tumor antigen and can regulate viral gene expression, can also heighten susceptibility to PML [6].

The primary risk factors for PML development appear to be the underlying disease itself and the immunosuppressive drugs administered. Several transcription factors associated with the replication of JCV, the causal agent of PML, are influenced by proinflammatory cytokines like tumor necrosis factor (TNF). B-cells also contribute to supporting JCV replication, which may explain the relatively elevated risk of PML observed in conditions such as chronic lymphocytic leukemia and other B-cell lymphoproliferative diseases. The transcription factor Spi-B, involved in B-cell differentiation and maturation, has been identified as a regulator of JCV gene expression [7]. Recombinase enzymes RAG1 and RAG2, responsible for rearranging immunoglobulin genes, have also been proposed as facilitators of JCV reactivation. In patients undergoing natalizumab treatment, which carries a high risk of PML [8], the mobilization of CD34+ hematopoietic precursor cells and CD19+ precursor B cells from the bone marrow has been suggested to facilitate JCV reactivation and dissemination [9]. However, even if such conditions facilitate JCV reactivation, the emergence of a neurotropic quasispecies is likely independent of disease-specific cofactors, as replication can proceed without obstruction from T cells [8,9].

Patients with rheumatic diseases, such as systemic lupus erythematosus (SLE), Wegener's granulomatosis, scleroderma, dermatomyositis, polymyositis, and rheumatoid arthritis, have also been more observed to develop PML [10]. That can be attributed to the use of immunosuppressive agents in their treatment, the underlying condition itself, or a combination of both factors. More recently, the association between PML development and biological drugs such as natalizumab for MS and rituximab for lymphoma and SLE has been established [11].

In individuals without underlying health conditions, the JCV is generally considered non-pathogenic, although approximately 30% of individuals intermittently excrete the virus in their urine [11]. After primary infection, the virus remains dormant in the kidneys, lymphoid organs, and bone marrow, where it is believed to be under immune system control [11]. The exact mode of infection is still unknown, but possibilities such as urine-to-oral transmission and respiratory spread have been suggested due to the presence of the virus in urine and tonsillar tissue, respectively [12]. Interestingly, during PML, the JC viral sequences detected in CNS tissues resemble those found in the blood and bone marrow, rather than in the kidneys or urine. This indicates that the virus-causing CNS disease likely originates from hematopoietic tissues [13]. The viral genome within the blood, bone marrow, and CNS contains tandem repeats in the transcriptional control region, a characteristic that has been demonstrated to be crucial for viral replication in glial cells in laboratory experiments [12,13].

The demyelination observed in PML occurs as a result of oligodendrocyte death primarily affecting subcortical white matter regions of the cerebral hemispheres [14], cerebellum, or brain stem. In immunosuppressed individuals (either due to intrinsic factors or medication-induced suppression), PML should be considered in the differential diagnosis when accompanied by neurological deficits. Additionally, a study by Lima et al. revealed that 18% of the reviewed PML patients experienced seizures as part of their symptoms [15].

PML can be diagnosed through both neuro-clinical and neuropathological approaches [16]. Neurologically, patients may present with progressive neurological symptoms that can be misdiagnosed as strokes. Magnetic resonance imaging (MRI), particularly T2-weighted sequences such as T2-FLAIR, is highly sensitive in detecting PML lesions and plays a crucial role in the diagnosis. Conventional T2-weighted spin-echo sequences can confirm the findings observed on T2-FLAIR and exhibit imaging characteristics that strongly suggest PML, aiding in the differential diagnosis [17]. Another diagnostic method involves detecting JCV DNA in CSF through PCR [17]. During the neuropathological examination, distinct histopathological features are observed, including demyelination, the presence of abnormal astrocytes, and the enlargement of oligodendroglial nuclei. To identify the presence of JCV in specific brain lesions, immunohistochemistry or electron microscopy can be utilized, whereas in situ hybridization or PCR can be employed to detect JCV DNA in those same lesions [17].

In cases of PML, CSF evaluation often appears normal, showing the absence of leukocytes, although there may be minimal CSF pleocytosis with a cell count of fewer than 20 cells/mL. Protein levels in the CSF are usually mildly elevated but remain below 100 mg/dL. The most reliable non-invasive test for confirming PML is PCR analysis of the CSF, which detects JCV-specific DNA [16,18]. This PCR-based diagnosis has reported a sensitivity of 75% and a specificity of 96%. Antibody testing is not reliable for determining active PML since a majority (86%) of the population has detectable levels of JCV-specific antibodies. In cases where CSF PCR evaluation yields negative results, detection of the JCV can be performed through in situ hybridization and/or immunohistochemistry on brain biopsies from suspected patients. Brain biopsies of PML lesions typically exhibit enlarged oligodendrocyte nuclei, which are characteristic of viral inclusion bodies, as well as oligodendrocyte cell death and abnormal JCV-infected astrocytes. Minimal inflammation accompanied by a mononuclear response is typically observed in brain biopsies of PML lesions, likely attributed to the immunosuppression of the patient [19].

Due to the lack of effective antiviral treatment against the JCV, the recovery of antiviral immune responses remains the only available treatment for PML. Recent therapeutic strategies aimed at promoting immune recovery have gained attention in PML management [20]. One approach involves the use of cytokines, such as interleukin-7 (IL-7), which has shown promise in non-controlled retrospective studies. Although survival did not significantly differ from expected outcomes in HIV/AIDS patients, there may have been improvements observed in individuals with hematological malignancies, primary immunodeficiencies, and transplant recipients. Administration of recombinant human IL-7 (rhIL-7) may contribute to increased blood lymphocytes and decreased JCV replication in CSF, which have been associated with better survival outcomes [21].

Another emerging strategy involves the adoptive transfer of anti-polyomavirus specific T cells. Studies have demonstrated the feasibility of generating polyomavirus-specific T cells from healthy related donors, and these cells can be safely infused for adoptive immunotherapy in patients with PML. Although the efficacy of this approach was not specifically evaluated, the data provide additional support for this potentially life-saving therapy, as some patients survived PML for more than one year after the initial infusion, while others succumbed to the disease within three months [22].

Additionally, immune checkpoint inhibitors (ICIs) have been investigated as a potential treatment for PML. However, non-controlled retrospective studies have shown high mortality rates among PML patients treated with ICIs. Furthermore, the development of inflammatory features or overt PML-IRIS (immune reconstitution inflammatory syndrome) was commonly observed. These findings underscore the importance of personalized treatment approaches with ICIs tailored to the specific characteristics of each PML patient [20].

## Conclusions

PML is a severe and often fatal disease, and despite its significance, there is limited research available that explores its association with multiple coexisting diseases and identifies the most relevant risk factors, particularly in the HIV-negative population. The similarity of neurological deficits between PML and cerebrovascular accidents can lead to misdiagnosis, and it may also present as new-onset seizures. Therefore, a high level of clinical suspicion is crucial for accurate diagnosis, which typically involves detecting viral DNA in CSF or through brain biopsy. There is a pressing need for extensive research to identify effective treatment options, which may need to be tailored to each individual case.
